# CHIPS-FF: Evaluating Universal Machine Learning Force Fields for Material Properties

**DOI:** 10.1021/acsmaterialslett.5c00093

**Published:** 2025-05-05

**Authors:** Daniel Wines, Kamal Choudhary

**Affiliations:** Material Measurement Laboratory, 10833 National Institute of Standards and Technology, Gaithersburg, Maryland 20899, United States

## Abstract

We introduce CHIPS-FF (Computational High-Performance Infrastructure for Predictive Simulation-based Force Fields), an open-source benchmarking platform for machine learning force fields (MLFFs). This platform focuses on complex properties including elastic constants, phonon spectra, defect formation energies, surface energies, and interfacial and amorphous phase properties. Utilizing 16 graph-based MLFFs including ALIGNN-FF, CHGNet, MatGL, MACE, SevenNet, ORB, MatterSim, and OMat24, CHIPS-FF integrates the Atomic Simulation Environment (ASE) with JARVIS-Tools to facilitate high-throughput simulations. Our framework is tested on a set of 104 materials, including metals, semiconductors, and insulators representative of those used in semiconductor components, with each MLFF evaluated for convergence, accuracy, and computational cost. Additionally, we evaluate the force-prediction accuracy of these models for close to 2 million atomic structures. By offering a streamlined, flexible benchmarking infrastructure, CHIPS-FF aims to guide the development and deployment of MLFFs for real-world semiconductor applications, bridging the gap between quantum mechanical simulations and large-scale device modeling.

## Graphical abstract

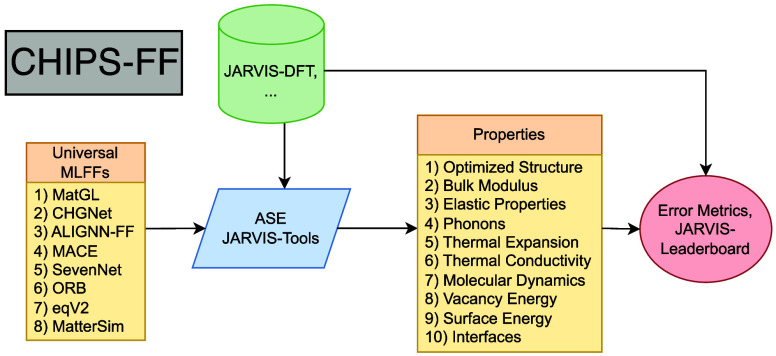

Multiscale modeling in materials science^1,2^ is an essential tool to bridge the gap between atomistic properties, device performance, and manufacturing. Recently, there has been a renewed focus on modeling semiconductor materials, devices, and components. Various approaches to semiconductor modeling can be applied at different scales, including quantum mechanical tools such as density functional theory (DFT),^3^ classical atomistic simulations such as molecular dynamics (MD),^4^ technology computer-aided design (TCAD)^5^ and various machine learning (ML) models.^6–12^ With the public availability of large DFT databases such as the Materials Project,^13^ Joint Automated Repository for Various Integrated Simulations (JARVIS)-DFT,^14,15^ Open Quantum Materials Database (OQMD),^16,17^ and Alexandria,^18–21^ several researchers have trained machine learning force fields (MLFFs) on full DFT databases (graph neural network-based), encompassing the entire periodic table. These so-called universal/unified/foundational machine learning force fields (uMLFFs) have been successful at reproducing near-DFT accuracy for a wide-range of systems.

Recently, interest in the field of uMLFFs has grown immensely (from academia, government and industry), as seen by the several models developed and various use cases of model architectures. Given the current state of the field, benchmarking the performance of uMLLFs are imperative. Large-scale efforts to benchmark the performance of various pretrained uMLFF architectures have emerged such as Matbench Discovery^22^ and the JARVIS-Leaderboard,^24,25^ which calculate error metrics and allow for models to be uploaded as time progresses. Matbench Discovery is an interactive leaderboard platform where uMLFFs are ranked based on accuracy of properties (such as energy and more recently thermal conductivity) and the ability to simulate high-throughput discovery of stable undiscovered inorganic materials. These uMLFFs are evaluated on targeted large-scale test data sets (i.e., a test set of 200,000 Materials Project entries) to calculate error metrics.^22^ The JARVIS-Leaderboard is a flexible benchmarking platform that allows for the evaluation of various machine learning, electronic structure, force-field and quantum computing, and experimental methods.^24,25^ In addition, there have been several focused benchmarking efforts of uMLFFs for more involved properties beyond energy.^26–31^ These recent efforts have highlighted the successes and limitations of various uMLFF architectures, and have emphasized the need for a comprehensive publicly available benchmarking platform. Such a platform allows the community to make informed decisions regarding uMLFFs, outweighing factors such as computational cost versus accuracy. In addition, researchers may find systematic trends in the uMLFF results which can help others to understand the applicability of each uMLFF type.

In this work, we present CHIPS-FF (Computational High-performance Infrastructure for Predictive Simulation-based Force Fields): a generalized user-friendly open-source benchmarking package to test various uMLFFs for a number of properties beyond energy. We have implemented a streamlined workflow to use uMLFFs for structural relaxation, bulk modulus, elastic properties, point defect formation energy, surface energy, interfacial properties (work of adhesion), molecular dynamics and creation of amorphous structures, all with automated error metrics from JARVIS-DFT. In contrast to large scale benchmarking efforts such as Matbench Discovery,^22^ which requires users to submit contributions covering large-scale test sets (on the order of 200,000), CHIPS-FF allows for robust benchmarking on smaller data sets and more complex properties. We intend this codebase and benchmarking data set to benefit the MLFF community and aid in the development of future models.

A majority of the reference data used to compare our uMLFF results to were obtained from the JARVIS-DFT database, which contains relaxed structural data (primitive lattice vectors, volume), elastic properties (bulk modulus, elastic tensor), phonon band structure,^14,15^ vacancy calculations^46^ and surfaces.^47^ All of the DFT data in JARVIS was computed with the vdW-DF-optB88 ^48^ functional. This is slightly different than other materials repositories, which mostly contain DFT calculations performed with the Perdew Burke Ernzerhof (PBE)^49^ functional. Despite comparing to JARVIS-DFT results (vdW-DF-optB88), the CHIPS-FF workflow has a flexible framework to use any DFT data set as a ground truth. Of course, comparing the results of a quantity computed with a uMLFF to a DFT result with an arbitrary exchange-correlation functional and varying convergence criteria may result in biased or inconclusive error metrics. In this case, it may be suitable to compare uMLFF results directly to available experimental data, especially if the uMLFF prediction is closer to “reality” than the DFT calculation it is being evaluated against.

Figure 1 depicts a full schematic of the CHIPS-FF workflow. In this workflow, initial structures are taken from the JARVIS-DFT database. JARVIS-Tools is used to pull structures from the JARVIS-DFT database and generate supercell surface and defect structures prior to atomistic simulations. Various uMLFF calculators are accessed through ASE. The uMLFFs tested in this work are shown in Table 1 and further details regarding the models and training data sets are given in the SI. As pretrained force fields are developed over time, they will be added to the workflow. In addition, CHIPS-FF offers the flexibility for the user to add their own force field or property to the pipeline. Once each uMLFF is loaded and run through the Atomic Simulation Environment (ASE)^50^ calculator, a variety of properties can be computed. As these calculations are carried out, detailed logging information is saved, including the computational timing for each stage of the calculation and whether or not each stage converged. Once these material properties are computed with each uMLFF, they are cross checked with entries in the JARVIS-DFT database and the mean absolute error (MAE) for each property is computed. In addition to automatically calculating the error metrics, our workflow will create entries for the JARVIS-Leaderboard in the appropriate format. From here, these entries can be directly uploaded to the leaderboard and compared to other benchmarks. Our CHIPS-FF package offers easy-to-use command line tools, which can easily be parallelized to simultaneously run hundreds or even thousands of materials/uMLFFs on CPU and GPU.

**Table 1:** Universal uMLFF Architectures Benchmarked in This Work^a^

**Model**	**Version**	**Submodel**	**Training data**
MatGL (M3GNet)^32–34^	1.1.2	M3GNet-MP-2021.2.8-PES	MPF
		M3GNet-MP-2021.2.8-DIRECT-PES	MPF
ALIGNN-FF^35^	2.12.2024	—	ALIGNN-FF DB
CHGNet^36^	0.3.8	—	MPtrj
MACE^37–39^	0.3.10	MACE-MP-0	MPtrj
		MACE-MPA-0	MPtrj+sAlex
		MACE-Alexandria^21^	Alex-2D^21^
SevenNet^40,41^	0.9.2	SevenNet-0 (11 Jul 2024)	MPtrj
ORB^42^	0.4.1	orb-v2	MPtrj+Alex
		orb-d3-v2	MPtrj+Alex
MatterSim^43^	1.0.1	MatterSim-v1.0.0–5M	MatterSim
OMat24 (eqV2)^44,45^	fairchem.core:	eqV2_31M_omat	OMat24
	1.2.1	eqV2_86M_omat	OMat24
		eqV2_153M_omat	OMat24
		eqV2_31M_omat_mp_salex	OMat24+MPtrj+sAlex
		eqV2_86M_omat_mp_salex	OMat24+MPtrj+sAlex

a Details of the submodels used and training datasets are depicted. Further details regarding the models and training datasets are discussed in the Supporting Information (SI).

**Figure 1:**
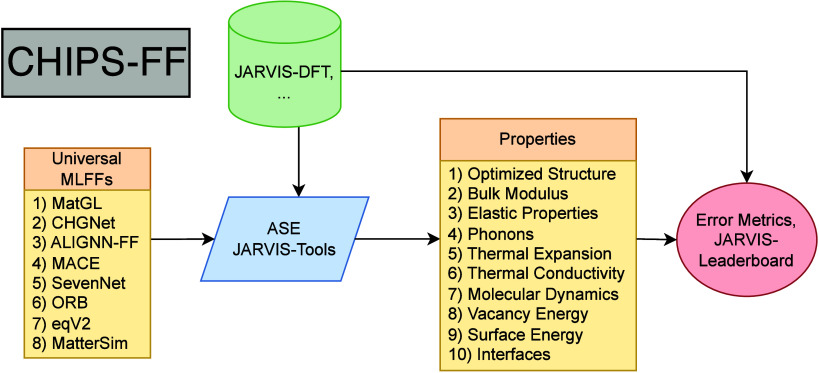
A full schematic of the CHIPS-FF workflow.

For our benchmarking study, we chose a set of 104 materials most commonly found in semiconductor devices and interfaces. This test set contains metals, semiconductors and insulators to be representative of the various parts and interfaces of integrated circuits. Figure S1 depicts various distributions of properties of the test set, including space group, band gap (calculated with vdW-DF-optB88), atomic number, chemical formula type, crystal system, and Wyckoff site, and dimensionality, all obtained from JARVIS-DFT. In addition, Table S1 and S2 give detailed information of the chemical composition, crystal structure and band gap for each material, where we see that the set contains 28 metals (zero band gap), 14 insulators (band gap above 3 eV) and 62 semiconductors (band gap between 0 and 3 eV). In addition, we see from Figure S1 that our test set represents a diverse set of materials ideal for testing our uMLFF workflow.

When considering the use of a pretrained uMLFF, the most important considerations should be accuracy, rate of convergence, scalability, and computational cost. After running the full CHIPS-FF workflow for the test set of 104 materials, we performed an analysis of which structural relaxation calculations reached convergence (force-maximum threshold of 0.05 eV/Å space within 200 steps) for bulk materials, surfaces and vacancies. This analysis is presented in Table 2 (FrechetCellFilter) and Table S3 (ExpCellFilter), where the percentage of unconverged results is depicted for each calculation type. Aside from ALIGNN-FF, all other uMLFF have a near-perfect convergence rate for bulk structures. With regards to surfaces and defects, the convergence rate is more variable when using the ExpCellFilter, but near-perfect when using the FrechetCellFilter (with the exception of ALIGNN-FF). Interestingly, the OMat24 models significantly vary in terms of convergence for surfaces depending on training data (for the ExpCellFilter). Specifically, the OMat24 models trained on OMat+MPTrj+sAlexandria have a significantly higher rate of convergence when compared to the models solely trained on OMat (see Table S3). These results (Table 2 vs Table S3) demonstrate the robustness of the FrechetCellFilter in ASE for these uMLFF models.

**Table 2:**
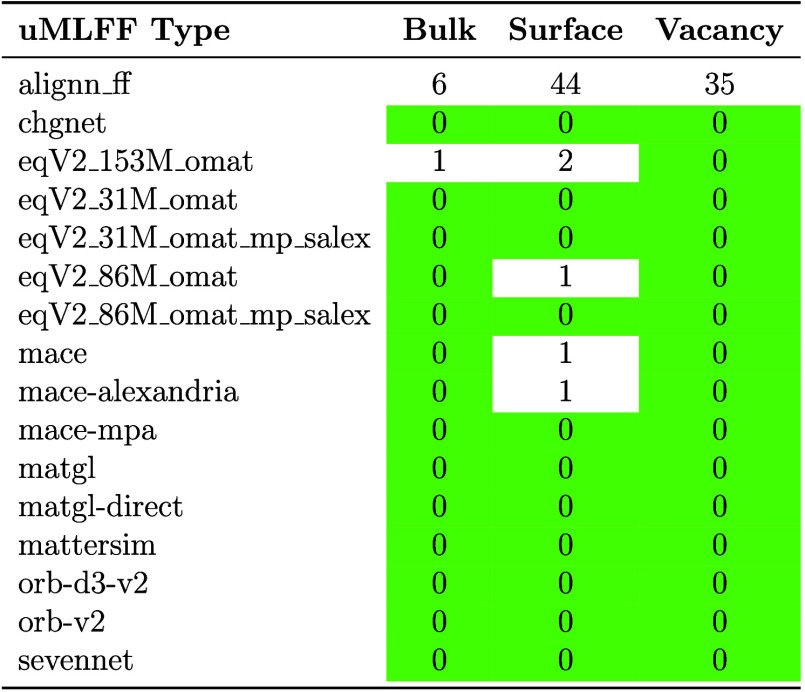
Percentage of Unconverged Structural Relaxations for Bulk, Surface, and Vacancy Calculations for Each uMLFF^a^

a Relaxed using the FrechetCellFilter.

For each of the 104 structures, we performed structural relaxations, fitting of the energy vs volume curve (equation of state), calculation of the elastic tensor, calculation of the phonon band structure, relaxation of all nonpolar surfaces (among [1, 0, 0], [1, 1, 1], [1, 1, 0], [0, 1, 1], [0, 0, 1], and [0, 1, 0]), and relaxation of each type of point defect in the material. The computational timing and errors in the lattice constants, and elastic properties for each uMLFF model are depicted in Table 3 (computed with the FrechetCellFilter). An interactive version of these results is available on the JARVIS-Leaderboard platform (see Figure 2b) for Pearson Correlation Coefficient metric), where it will be continuously updated as uMLFF models are developed and released. In addition, structural results computed using the ExpCellFilter are depicted in Table S4. Unsurprisingly, we observe more accurate lattice parameters computed with the FrechetCellFilter due to its robustness in optimizing structural parameters (see Table 3 vs Table S4). In Table 3, we see that all uMLFF models are able to compute reasonably accurate structural parameters. ALIGNN-FF does an excellent job of simultaneously capturing a, b, and c. This is expected due to the fact that ALIGNN-FF was trained on the JARVIS-DFT data set (vdW-DF-optB88) and the target/“ground truth”, in addition to the initial structures, are from JARVIS-DFT. The OMat24 models perform exceedingly well, but are the most computationally expensive. The ORB models also perform exceedingly well for structural relaxation and have the advantage of being close to an order of magnitude more computationally efficient. One interesting trend we observe in our results for this set of 104 materials is that the error for lattice constant c is substantially higher than a and b. This can be due in part to the test set containing a significant portion (≈ 10%) of vdW bonded (“2D-bulk-like”) layered structures (see Figure S1). Since most of these uMLFF were trained on PBE data without vdW corrections (with the exception of ALIGNN-FF), we can expect larger errors for vdW systems. For example, we observe significant errors for hexagonal BN (JVASP-62940) when relaxed with orb-v2, where we observe over 10% error in c. These errors are drastically more significant when using the ExpCellFilter along with orb-v2, where we observed a 130% error in c for JVASP-62940 (see Table S4 for total MAE). Fortunately, some of these models such as MACE and ORB have explicit dispersion corrections that can be added to more accurately address vdW interactions.^37–39,42^ To test this, we ran the workflow for dispersion-corrected orb-d3-v2. For orb-d3-v2, we observe a reduction in error for c and volume (see Table 3). For JVASP-62940 relaxed with orb-d3-v2, the percent error in c is reduced to 0.02%. This emphasizes the importance of dispersion corrections in uMLFF architectures for vdW bonded materials.

**Figure 2:**
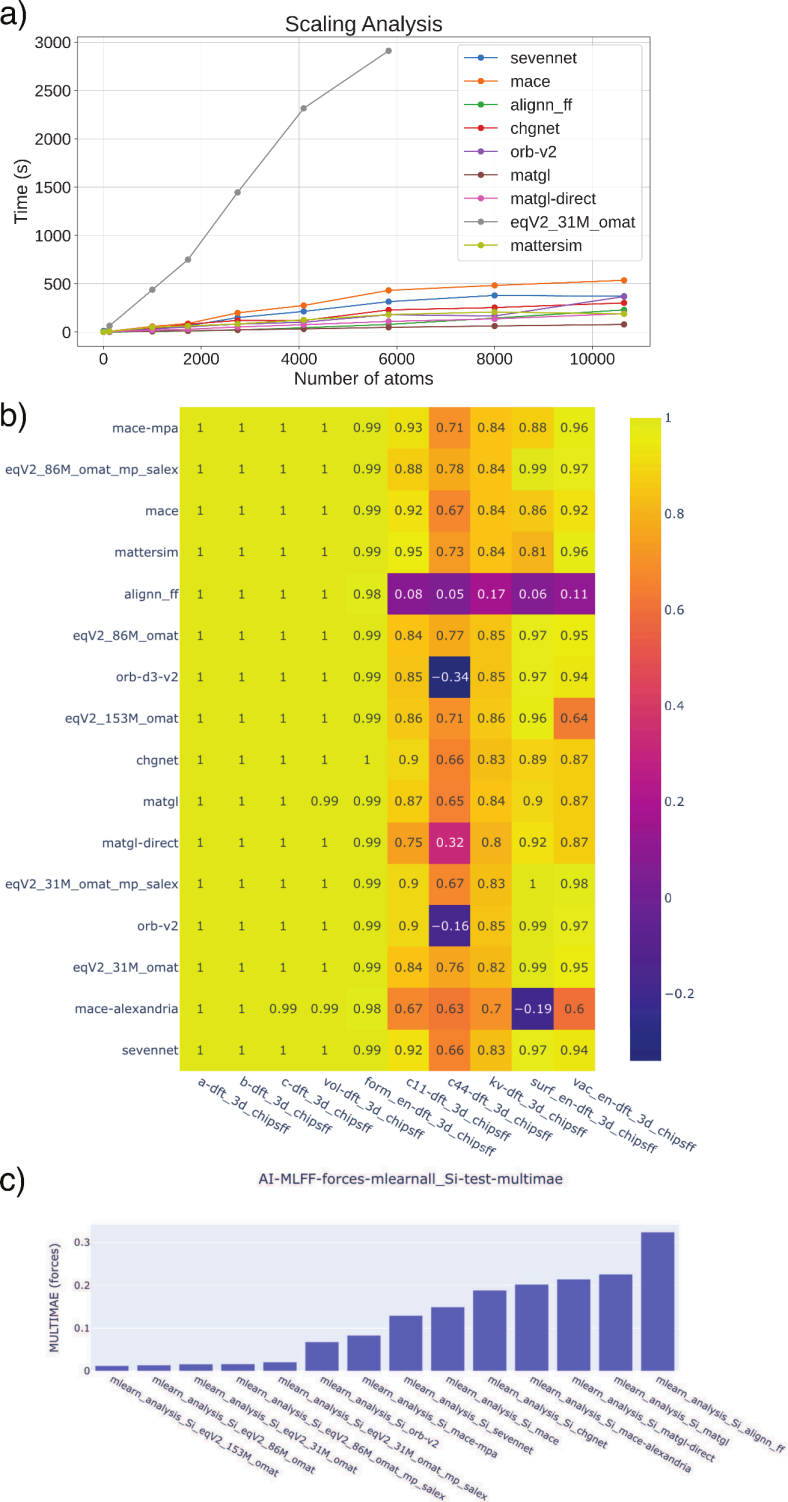
a) Scaling analysis of various uMLFF up to 10,000 atoms (for a supercell of Cu), b) an example JARVIS-Leaderboard entry for the force MAE of the MLEARN data set, c) an example of the interactive error metrics within the JARVIS-Leaderboard (Pearson correlation coefficient).

**Table 3:**
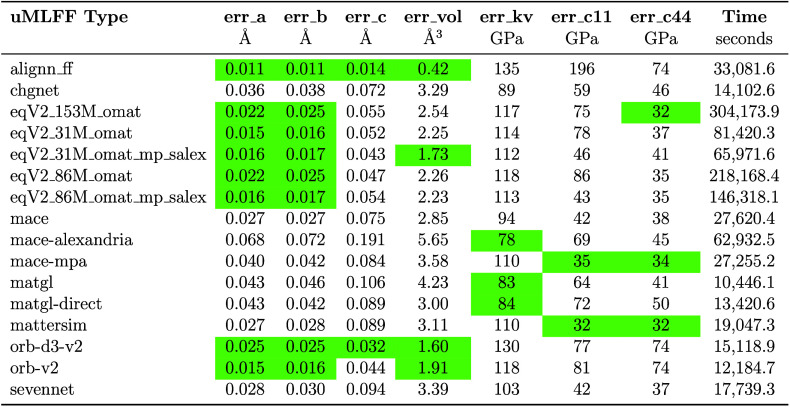
Mean Absolute Error (MAE) and Total Computational Time for a Variety of Material Properties Calculated with Each uMLFF Type^a^

a Relaxed using the FrechetCellFilter. The MAE is calculated with respect to JARVIS-DFT data. Properties include (in order) lattice constants a, b, c, volume, bulk modulus, and C11 and C44 components of the elastic tensor. The computational time is measured per CPU. An interactive and up-to-date version of the table is available in the JARVIS-Leaderboard. The green indicates the best performing models.

Fitting the equation of state to obtain bulk modulus and calculating the elastic tensor prove to be a more challenging task for these uMLFF models, emphasizing difficulties in modeling the potential energy landscape far from equilibrium. We found that models such as MACE-Alexandria and MatGL have superior performance in predicting the energy vs volume curves compared to the OMat and ORB models. For contributions to the elastic tensor (C11 and C44), we also observe relatively high errors. We see that only a few models can simultaneously predict C11 and C44 with reasonable accuracy (MAE less than 50 GPa). Interestingly, we found that eqV2_31M_omat, eqV2_86M_omat and eqV2_153M_omat can predict C44 with reasonable accuracy, but have much higher prediction errors for C11, while eqV2_31M_omat_mp_salex and eqV2_86M_omat_mp_salex do a better job at predicting C11 and C44 simultaneously. We observe that MACE-MPA-0 and MatterSim give the best results for simultaneous C11 and C44 predictions.

Out of the 104 materials in our test set, there were 84 matching entries for DFT phonon calculations in JARVIS, from which the composite MAE of the phonon band structure was computed in Table 4. We performed these phonon calculations with the uMLFFs (along with phonopy^51,52^ ) at various displacements in the finite-displacement calculation of force constants. We found that models such as ALIGNN-FF and MatGL have relatively large errors in phonon predictions. For certain uMLFFs, we observe a consistent phonon MAE as the displacements get smaller (MACE-MP-0, MACE-MPA-0, MACE-Alexandria, Mattersim, SevenNet). For ORB and OMat models, we see a drastic increase in phonon MAE as the displacements become smaller. This signifies that ORB and OMat models could possibly perform poorly in the low-force regime due to increased noise from the small displacements. This is consistent with recent results, where it was reported that OMat and ORB models had substantial errors in phonon predictions (for small displacements) when compared to models such as MatterSim, MACE, CHGNet, and SevenNet for 10,000 materials.^31^ This work hypothesized that the errors are due to ORB and OMat computing forces as a direct output of the neural network as opposed to calculating the forces by evaluating the derivative of the energy with respect to the atomic positions (through back-propagation).^31^ MatterSim offers similar performance (and consistency across atomic displacements) in terms of predicting the phonon band structure when compared to accurate equivariant uMLFFs such as MACE and SevenNet. This is especially interesting since MatterSim-v1 is not an equivariant uMLFF. In addition, we see improved phonon results for the newer MACE-MPA-0 when compared to MACE-MP-0.

**Table 4:** MAE for Phonon Band Structure (in cm^–1^) Computed with Different Values of Displacement

	**Displacements (Å)**			
**Method**	**0.2**	**0.05**	**0.01**	**0.001**
alignn_ff	155	155	157	164
chgnet	70	75	83	91
eqV2_153M_omat	49	47	49	75
eqV2_31M_omat	48	46	53	73
eqV2_31M_omat_mp_salex	48	46	112	219
eqV2_86M_omat	51	47	49	77
eqV2_86M_omat_mp_salex	48	46	103	208
mace	58	60	60	60
mace-alexandria	82	84	85	85
mace-mpa	50	50	50	50
matgl	82	93	94	94
matgl-direct	86	101	102	101
mattersim	48	47	47	47
orb-d3-v2	53	50	85	181
orb-v2	49	50	108	201
sevennet	54	55	56	56

In addition to summing the computational timing for the entire workflow, we also examined the scaling behavior of each uMLFF (on CPU). These results for supercells of Cu (up to 10,000 atoms) are depicted in Figure 2a). Unsurprisingly, we see that OMat model scales much more drastically with system size as compared to the other uMLFF models. In addition, we found that the invariant models have more favorable scaling than the equivariant models such as MACE and SevenNet, with SevenNet having slightly better scaling than MACE. The scalability of ORB and MatterSim is a huge advantage due to their accuracy of various material property predictions. Similar scalability studies were conducted in ref.^42^ The quantities of surface energy and vacancy formation energy are difficult to predict with machine learning methods and provide a rigorous benchmarking test for uMLFF architectures. Out of the 104 materials, there exists 85 entries for surface DFT calculations and 48 entries for defect DFT calculations within JARVIS-DFT. We used these calculations to compare our uMLFF results. Figure 3a) and Figure 3b) depict the parity plots and corresponding MAE for surface energy and vacancy formation energy of our test sets. The very low error for surface energy (0.16 J/m^2^) and vacancy formation energy (0.36 eV) highlight some of the successes of recent state-of-the-art models such as OMat24, ORB, MACE-MPA-0 and MatterSim. We also observe reasonably accurate results for MACE-MP-0 and SevenNet when predicting vacancy formation energy and surface energies. Recently, the proprietary model PFP from Preferred Networks Inc. was evaluated for our CHIPS-FF surface energy benchmark and achieved an MAE of 0.19 J/m^2^,^53^ achieving similar accuracy to the OMat24 and ORB models. The low cost of ORB, high rate of convergence and high accuracy make it a viable tool to relax larger surfaces and defect supercells.

**Figure 3:**
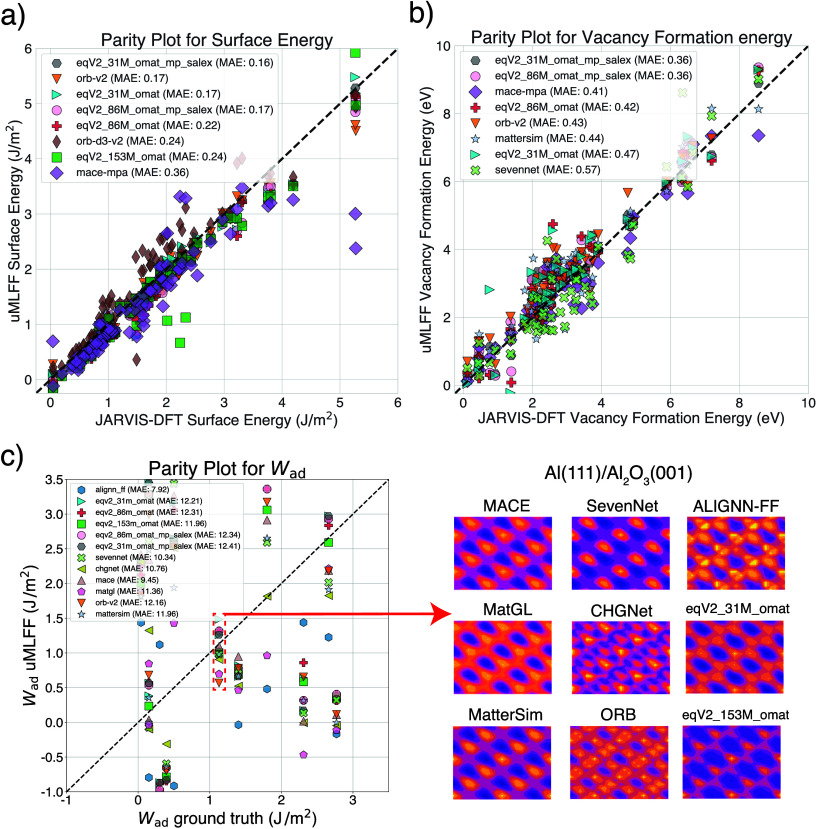
Parity plots for a) surface energy (in J/m^2^) and b) vacancy formation energy (in eV) for each uMLFF type with respect to JARVIS-DFT data (MAE is given in the inset and the best performing models are included in the plots). c) Depicts the parity plot for work of adhesion (W_*ad*_ in J/m^2^) for several material interfaces and MAE with respect to previous calculated and experimental data (taken from ref 54). The parity plot is zoomed in to focus on a subset of calculations, but the MAE is computed for the entire test set. To the right of the parity plot, we focus on the best performing W_*ad*_ prediction for the Al(111)/Al_2_O_3_(001) interface, showing the xy grid search and optimal in-plane interface configurations with each uMLFF.

In addition to benchmarking the properties of bulk materials, we utilized the CHIPS-FF workflow for interface calculations (along with the InterMat^47^ package). Figure 3c depicts the parity plot for work of adhesion (W_*ad*_) for various material interfaces. The MAE for W_*ad*_ is computed with respect to “ground truth” data taken from ref.^54^ (from experiment and theory). As seen in Figure 3c, predictions for W_*ad*_ are extremely poor for each uMLFF type. The parity plot in Figure 3c is zoomed in to focus on a subset of calculations, but the MAE is computed for the entire test set of interfaces, which include significant outliers. This is not surprising due to the fact that none of these uMLFF are trained on interface data. We went on to analyze one of the best performing predictions for an Al(111)­Al_2_O_3_(001) interface (see red box on Figure 3c). From here, we performed an in-plane (xy) grid search to find the optimal energy configuration of the interface. In addition, this in-plane scan can be a test of how smooth the potential energy surface is. On the right panel of Figure 3c we see the results of this interface scan for a number of uMLFFs. We observe a smoother potential energy surface for MACE, SevenNet, MatterSim and OMat24 models. Although the MAE is extremely high for all uMLFF models for W_*ad*_, it is the lowest for ALIGNN-FF. This can be due in part to ALIGNN-FF being trained on vdW-corrected DFT calculations and having a substantial amount of exfoliable materials in the data set.

We chose to benchmark the performance of uMLFF models for the generation of amorphous Si (a-Si) by performing melt/quench simulations. Figure 4 depicts a summary of these results, where the radial distribution function (RDF) is shown as a function of pair separation distance. We benchmarked these uMLFF RDF curves against the RDF curve obtained from computationally expensive AIMD simulations for a-Si. The inset of Figure 4 depicts the MAE of each RDF curve with respect to AIMD. We observe that architectures such as MACE, SevenNet, and OMat24 (eqV2), CHGNet, ALIGNN-FF, MatterSim and ORB have excellent accuracy, while MatGL has a slightly higher MAE and a lower R^2^ value. The final amorphous structures for AIMD, MatterSim, eqV2 and ORB (best performing uMLFF models) are given in the inset of Figure 4. The exceptional performance of ORB and MatterSim (both invariant uMLFFs) for a-Si is significant due to the fact that it is much less costly than other similarly performing equivariant uMLFF models. The relatively high accuracy of ALIGNN-FF with regards to a-Si is surprising due to the fact that ALIGNN-FF had inferior performance for a number of properties and is also an invariant model. A more thorough assessment of how ALIGNN-FF and other uMLFFs can model amorphous materials will be the subject of future work, which will also utilize the CHIPS-FF infrastructure. These results bring into question whether or not equivariance is necessary to achieve accurate results for amorphous materials with uMLFFs, and more rigorous benchmarking will need to be conducted to bring clarity to this.

**Figure 4:**
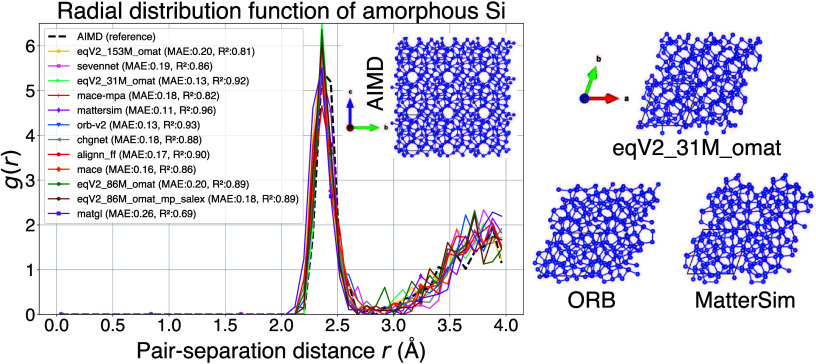
Calculated radial distribution function (*g*(*r*)) as a function of pair separation distance computed with several uMLFF models for amorphous Si (by performing melt/quench simulations). Ab initio (AIMD) results are given as a ground truth benchmark (black dotted line) and the MAE and R^2^ with respect to AIMD is reported in the inset. The atomic structure for amorphous Si is given for AIMD and the best performing uMLFF models (MatterSim, eqV2_31M_omat and ORB).

In addition to testing these uMLFF models on a smaller and more focused data set, we also tested on a larger and more diverse data set for force predictions. Unlike total energy, forces are more independent of exchange-correlation functional and the accuracy of force predictions on various data sets can give valuable insight into how a particular model performs. We chose to benchmark each uMLFF on the MLEARN data set from ref.,^55^ which consists of face-centered cubic (Cu, Ni) and body-centered cubic (Li, Mo) metals and diamond group IV semiconductors (Si, Ge) which span a wide range of crystal structures and bonding environments (≈ 200–300 data points for each element). These results are depicted in Table 5. In addition, we benchmarked the accuracy of force predictions on very large data sets that were used to train uMLFFs. These data sets included ALIGNN_FF_DB (307,000 used to train ALIGNN-FF), MPF (188,000 used to train M3GNet), and MPTrj (1.58 million used to train CHGNet, MACE, SevenNet and used in the training of ORB and OMat models). These results are depicted in Table 6. Unsurprisingly, superior accuracy is obtained across all data sets when the OMat and ORB models are used to predict forces. We also observe highly accurate forces for MatterSim and a substantial reduction in force error when going from MACE-MP-0 to MACE-MPA-0. The CHIPS-FF package has built in functions to perform these benchmarking calculations on each respective data set, with a flexible framework to add additional data sets (such as OMat and Alexandria) and updated uMLFFs as they develop over time.

**Table 5:**
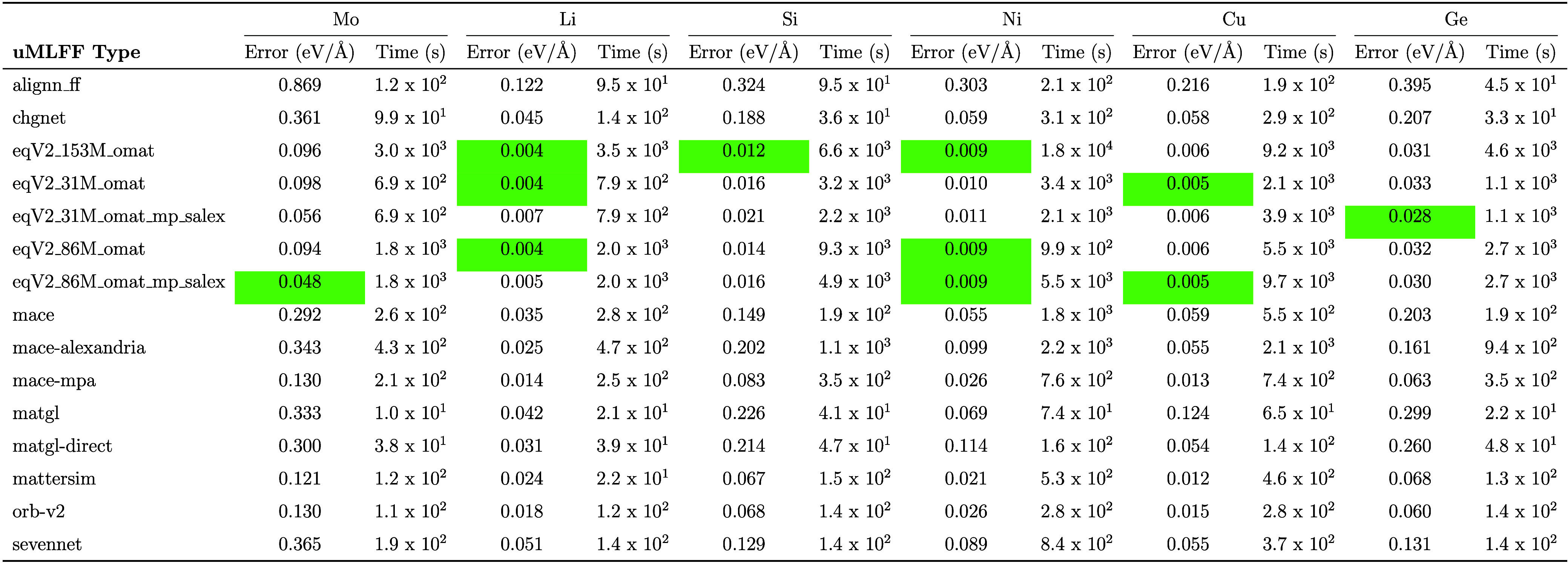
A Comparison of Force Errors (eV/Å) and Timings (s) for the mlearn DFT Dataset for Mo, Li, Si, Ni, Cu, and Ge^a^

a The green color highlights the best performing models for each element.

**Table 6:**
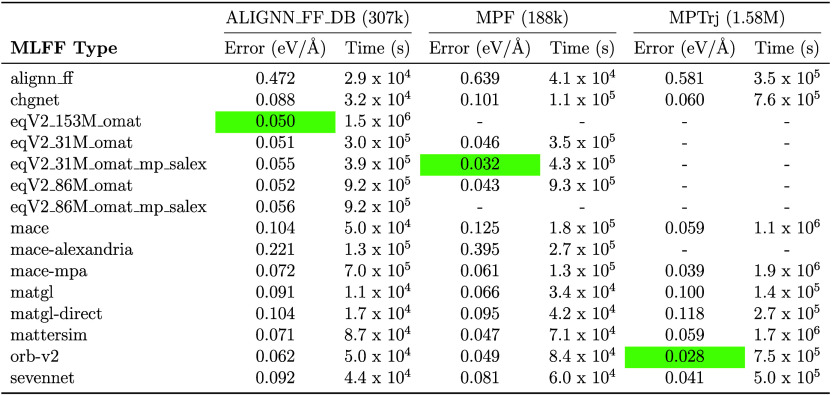
A Comparison of Force Errors (eV/Å) and Timings (s) for Different Datasets: ALIGNN_FF_DB, MPF, and MPTrj^a^

a The green color highlights the best performing model.

We have introduced CHIPS-FF, an open-source benchmarking platform for MLFF architectures, which has the capability to test properties beyond the standard total energy, including forces, phonons, elastic properties, surface energy, vacancy formation energy and properties of interfaces. We benchmarked several recent state-of-the-art uMLFF architectures on a subset of 104 materials most relevant for the semiconductor industry, taking into account accuracy with respect to DFT, convergence and computational cost. As MLFFs continue to develop over time, we expect the CHIPS-FF benchmarking platform to be critical in terms of testing the quality of uMLFFs.

## Supplementary Material



## Data Availability

The CHIPS-FF package can be found at https://github.com/usnistgov/chipsff. Related benchmarks for this work can be found at https://pages.nist.gov/jarvis_leaderboard/Special/CHIPS_FF/.

## Notes

Review of machine learning force field architectures, detailed methodology, description of software used, detailed description of test set, results for ExpCellFilter, CHIPS-FF tutorial (PDF)
