# Supplementary material for: CHIPS-FF: Evaluating Universal Machine Learning Force Fields for Material Properties
Source: ACS Mater Lett. 2025 May 5;7(6):2105–14. doi: 10.1021/acsmaterialslett.5c00093 (PMC12135062; doi:10.1021/acsmaterialslett.5c00093)
Supplement: Supplementary file 1 [file tz5c00093_si_001.pdf]

Supporting Information: CHIPS-FF: Evaluating Universal Machine Learning Force Fields for Material Properties

Daniel Wines* and Kamal Choudhary

Material Measurement Laboratory, National Institute of Standards and Technology, Gaithersburg, MD 20899, USA

E-mail: daniel.wines@nist.gov

Review of Machine Learning Force Fields

One of the pioneering machine learning force field (MLFF) architectures was developed by Behler and Parrinello in 2007 using neural networks.[1] Other MLFF methods include Gaussian processes-based Gaussian approximation potentials (GAP),[2] spectral neighbor analysis potential (SNAP)[3] and Allegro.[4] Graph neural network (GNN)-based machine learning force fields (MLFFs) are now considered state-of-the-art due to their superior accuracy and transferability. One of the first graph-based universal MLFF (uMLFF) architectures introduced in 2022 was M3GNet from Chen and Ong.[5] M3GNet has since transformed into MatGL (Materials Graph Library),[6] a re-implementation of M3GNet built on the Deep Graph Library (DGL)[7] and PyTorch.[8] M3GNet evolved from the MEGNet (MatErials Graph Network) property prediction model introduced in Ref.[9] M3GNet was trained on ≈ 60,000 inorganic materials (≈ 190,000 relaxation steps) in the Materials Project (MP), covering 89 elements of the periodic table.[5] This dataset is commonly referred to as MPF.[5] In M3GNet, the an-

gles (representing three-body interactions) are incorporated by aggregating to bonded atoms within the graph convolution steps to update atoms, bonds and properties.[5] The Atomistic LIne Graph Neural Network (ALIGNN)-FF model[10] was introduced in 2022 and was trained on the JARVIS-DFT dataset of $\approx$ 75,000 materials ($\approx$ 300,000 relaxation steps, ALIGNN-FF DB). ALIGNN-FF evolved from the ALIGNN property prediction model released in 2021.[11] The ALIGNN model achieved significant improvement in property prediction tasks due to the inclusion of bond-angles in terms of line graphs, which previous GNN models lacked.[11] The earlier versions of ALIGNN-FF utilized a k-nearest neighbor graph,[10] while the updated version of ALIGNN-FF (2024.12.2) utilizes a radius graph, making it faster and more robust. Another uMLFF that utilizes a crystal graph (atom graph and bond graph) is the Crystal Hamiltonian Graph Neural Network (CHGNet),[12] which was proposed in 2023. This model was trained on the Materials Project Trajectory (MPtrj) dataset,[12] which includes DFT calculations for $\approx$ 150,000 materials ($\approx$ 1.58 million relaxation steps). CHGNet also incorporates magnetic moments into the training process to enhance the description of chemical reactions.[12]

MACE,[13–15] an equivariant message passing neural network potential, uses higher order messages (higher than two body) combined with atomic cluster expansion (ACE),[16,17] which is a method for deriving an efficient body-ordered symmetric polynomial basis to represent functions of atomic neighborhoods. A pre-trained model for the entire periodic table (trained on MPTrj) for MACE was released in late 2023[15] (referred to as MACE-MP-0). In late 2024, a pretrained version of MACE (known as MACE-MPA-0) was released, which is trained on a combination of MPTrj and a subsampling of Alexandria (sAlex).[18] SevenNet (Scalable EquiVariance Enabled Neural Network),[19] another equivariant message passing uMLFF, is based on the NequIP[20] architecture for building E(3)-equivariant force fields. SevenNet trained on the MPtrj dataset and was released in 2024. In addition to these open source models, there have been proprietary uMLFF models developed including MatterSim (Microsoft),[21] GNoME (Google DeepMind),[22] and PFP (Preferred Networks

Inc).[23] A version of MatterSim (MatterSim-v1), which has a similar but slightly modified architecture to M3GNet and is trained on over 17 million structures sampled from Materials Project, Alexandria, and Microsoft's own dataset (consisting of additional structures and MD trajectories at ambient and extreme conditions), was made open source to the community in December 2024.[24] The Orb[25] pretrained uMLFF, released in fall 2024, was developed by the startup company Orbital Materials, where superior performance for force and energy predictions were demonstrated at significantly reduced computational cost.[26–28] Orb utilizes an attention augmented Graph Network-based Simulator (GNS),[29] which is a type of Message Passing Neural Network (MPNN).[30] In contrast to equivariant models such as MACE and SevenNet, Orb does not utilize equivariant message passing. The open source Orb model (orb-v2) is trained on MPtrj and Alexandria (over 3 million materials and 32 million relaxation steps).[26] In late 2024, Meta FAIR Chemistry released the Open Materials 2024 (OMat24)[31] models and dataset, which contains over 110 million DFT calculations and pretrained uMLFF models based on the EquiformerV2[32] architecture, which is a highly accurate equivariant transformer model. OMat24 offers various sized models trained on OMat, MPTrj and sAlex.

Methods

CHIPS-FF mainly relies on several Python-based packages: Atomic Simulation Environment (ASE),[33] JARVIS-Tools,[34,35] phonopy,[36,37] elastic[38–40] and Intermat.[41] These packages work in conjunction to design and manipulate atomic structures, access materials databases and compute physically observable quantities. ASE is a general toolkit for setting up, running and analyzing atomistic calculations for a variety of electronic structure and molecular dynamics codes. In addition, it allows for the integration of machine learning force fields (MLFFs) as calculators within the atomistic workflows. JARVIS-Tools, which utilizes ASE for certain functionalities, is an infrastructure to perform high-throughput atomistic calcu-

lations, manipulate structures, and parse data and results from different electronic structure and molecular dynamics codes. The main feature of JARVIS-Tools is the seamless integration of large-scale materials databases, such as JARVIS-DFT, which can be used to generate initial structures, filter materials based on specific properties and compare data with density functional theory (DFT) benchmarks. In this work, we utilize the JARVIS-DFT datasets of bulk materials, surfaces, and defects. The phonopy package is used to determine phonon and lattice dynamics based on computed force constants, which can be obtained from the finite-displacement method. These force constants can be computed via DFT calculations or via MLFF. The phonon dispersion, phonon density of states and derived properties such as heat capacity and zero-point energy can be calculated from phonopy. The elastic package (which utilizes routines in ASE) is based on standard elasticity theory and the finite deformation approach to compute the elastic tensor of a crystal. The Intermat package is a toolkit for the generation of material interfaces that utilizes ASE and JARVIS-Tools to carry out high-throughput simulations. Interface configurations can be generated using the algorithm of Zur et al.[42] and the x-y (in-plane) and z (out-of-plane) parameters of the interface can be optimized with DFT or MLFFs (passed through ASE).

In terms of pretrained uMLFF models, we utilized ALIGNN-FF: 2.12.2024, CHGNet: 0.3.8, MatGL: 1.1.2 (using both the M3GNet-MP-2021.2.8-PES and M3GNet-MP-2021.2.8-DIRECT-PES[43] models), MACE: 0.3.10 (using MACE-MP-0 and MACE-MPA-0), SevenNet-0: 0.9.2 (using the 11July2024 model), orb-models: 0.4.1 (orb-v2 and orb-d3-v2, which utilizes the D3 dispersion correction[44]), and MatterSim-v1 (MatterSim-v1.0.0-5M). In addition, we benchmarked a MACE force field trained on the Alexandria dataset presented in Ref.[45] (this model was trained on over 200,000 2D material calculations with the PBEsol[46] functional and is referred to as MACE^{2D} in that work[47]). In our work, we refer to this model as mace-alexandria (which is distinct from mace-mpa). For the OMat24 models from Meta, we tested the three EquiformerV2 (eqV2) models trained on OMat (Small: 31M (million), medium: 86M, and large: 153M in terms of model parameters) and two

eqV2 models trained on OMat+MPTrj+Alexandria (Small: 31M and medium: 86M).[31,48] These models are named as: eqV2_31M_omat, eqV2_86M_omat, eqV2_153M_omat, eqV2_31M_omat_mp_salex, and eqV2_86M_omat_mp_salex.[31,48]

Initial structures were obtained from the JARVIS-DFT database (but not limited by it) and then optimized using the FIRE[49] algorithm within ASE. For bulk relaxations, atomic coordinates and lattice vectors were allowed to relax, but for surfaces and vacancy calculations, cell volume was fixed and atomic positions were allowed to relax. The maximum force value (stopping criterion for relaxation) was set to 0.05 eV/Å. If a calculation did not reach the stopping criterion by 200 steps, it was considered unconverged. If a structure did not reach convergence within 200 steps, the final structure and energy at 200 steps was logged and used for subsequent portions of the workflow. We tested both the `FrechetCellFilter` (main text) and `ExpCellFilter` (in SI) within ASE.[50] The bulk modulus was obtained by applying isotropic strain to the system from -6 % to +6 % and using a Murnaghan fit for the equation of state. The elastic tensor was computed using the Elastic package.[38–40] For bulk modulus and elastic tensor calculations, the conventional cell was used. We utilized the phonopy[36,37] package to perform simulations of the phonon spectrum using the finite displacement method,[51] where a 2 x 2 x 2 supercell was constructed from the initial unit cell. Displacement values of 0.2 Å, 0.05 Å, 0.01 Å, and 0.001 Å were benchmarked. From these phonopy results, we also extracted the Helmholtz free energy (and zero point energy at 0 K), entropy and heat capacity as a function of temperature. We have added capabilities to CHIPS-FF to compute the thermal expansion of each material at constant pressure and the lattice thermal conductivity, utilizing the quasi-harmonic approximation[52] implemented in phonopy and phono3py[36,37] respectively (these simulations are ongoing and will be discussed in future work). For spectral quantities such as the phonon band structure, we computed the MAE for each band along the q-point path using:

$$\mathrm{MAE} = \frac{1}{N_q} \sum_{q\nu} \left| \omega_{q\nu}^{\mathrm{uMLFF}} - \omega_{q\nu}^{\mathrm{DFT}} \right| \tag{1}$$

where $\omega_{q\nu}$ is the phonon energy (in cm^{-1}) at each q-point and each branch index (ν) (calculated with uMLFF and DFT from JARVIS-DFT) and N_q represents the number of wavevectors along the q-path. JARVIS-Tools was used to automatically generate supercells for neutral point defects (vacancies) and non-polar surfaces (both of which were later relaxed with uMLFFs). In this work, we obtained initial defect structures from the relaxed structures in the JARVIS-DFT vacancy database from Ref.[53] This allowed us to make a 1:1 comparison between vacancy formation energies (calculated with DFT and uMLFFs) for the exact same crystal structure and supercell size (avoiding finite-size scaling effects). In Ref.,[53] these defect supercells were constructed from the conventional cell and enforced to have a lattice vector c of at least 8 Å , where single vacancies were created based on Wyckoff-position information for each atomic species in the crystal. The following formula is used to obtain the vacancy formation energy (E_{vac}):

$$E_{\mathrm{vac}} = E_{\mathrm{defect}} - E_{\mathrm{bulk}} + \mu \tag{2}$$

where E_{defect} is the total energy of the defect supercell, E_{bulk} is the energy of the bulk structure (no defect) and μ is the chemical potential of the missing atom. The lowest energy crystal structure of the elemental solid (from JARVIS-DFT) is used to calculate the chemical potential with each respective uMLFF (where the structure is fully re-relaxed). Surfaces were generated for miller indices of [1, 0, 0], [1, 1, 1], [1, 1, 0], [0, 1, 1], [0, 0, 1], and [0, 1, 0], skipping any polar surfaces in the surface relaxation. Each surface contained at least 4 layers and 18 Å of vacuum. The surface energy of each non-polar surface was calculated as:

$$\gamma = \frac{E_{\mathrm{surface}} - N \cdot E_{\mathrm{bulk}}}{2A} \tag{3}$$

where E_{surface} is the energy of the surface structure, E_{bulk} is the energy of the bulk structure, N is the number of bulk unit cells in the surface, and A is the cross sectional area of the surface. In order to study amorphous materials, we have implemented finite-temperature

molecular dynamics into our workflow to perform melt/quench simulations using Berendsen NVT (Number of particles, Volume, Temperature ensemble) dynamics within ASE. For our case study of amorphous Si, we used a timestep of 1 femtosecond and ran the simulation at 3500 K for 10 ps (melt) and 300 K for 20 ps (quench). For benchmarking calculations to compare uMLFF results to, we performed similar ab initio MD (AIMD) calculations with the Vienna Ab initio Simulation Package (VASP) code, using projector augmented wave (PAW) pseudopotentials[54,55] and the vdW-DF-optB88 functional. These gamma-point AIMD calculation used a plane wave cutoff energy of 500 eV and the Nosé-Hoover[56–58] thermostat to run NVT simulations at 2000 K for 5 ps (melting), and 300 K for 5 ps (at a timestep of 1 femtosecond). In order to perform calculations for material interfaces, we utilized InterMat,[41] a package for the generation and calculation of interface structures (substrate plus film). The initial interface is obtained by creating a superlattice or alternating slab junction (ASJ) structure (without vacuum padding)[59,60] and then using the Zur algorithm[42] to obtain the best candidate interface. From here, we computed the relative alignment between the film and substrate in the in-plane (xy) direction by performing a grid search with a 0.05 fractional spacing interval with each respective uMLFF model. In addition to determining the optimal in-plane orientation between the substrate and film, this gives us a qualitative estimate of how smooth the potential energy surface is at the interface for each pretrained uMLFF. From this calculation, we also determined the work of adhesion (W_{ad}) at the interface with:

$$W_{\text{ad}} = \gamma_{\text{film}} + \gamma_{\text{substrate}} - \gamma_{\text{interface}} \tag{4}$$

where γ_{film} is the surface energy of the film, $\gamma_{\text{substrate}}$ is the surface energy of the substrate and $\gamma_{\text{interface}}$ is the interfacial energy calculated as:

$$\gamma_{\text{interface}} = \frac{E_{\text{interface}} - E_{\text{film}} - E_{\text{substrate}}}{A} \tag{5}$$

where $E_{\text{interface}}$ is the total energy of the interface, E_{film} is the total energy of the film,

$E_{\text{substrate}}$ is the total energy of the substrate and A is the cross sectional area.

Although it is possible to assess the accuracy of MLFFs by comparing results directly to DFT or experiment, there is a lack of explicit uncertainty quantification (UQ) for most MLFFs, including the universal pretrained MLFFs mentioned in this work. A robust UQ method should account for uncertainty arising from measurement noise (aleatoric) and uncertainty in predictions arising from model error (epistemic). Due to the fact that the DFT training data does not possess aleatoric error, most of the uncertainty for MLFFs is epistemic, which can be due to scarcity of data, limitations of model architecture, or poor parameter optimization within the model. Recently, different epistemic UQ methods such as ensemble-based uncertainty,[61] deep evidential regression,[62] mean-variance estimation,[63] Gaussian mixture models[64] have been implemented and benchmarked for atomistic MLFFs.[65,66] It is evident that low-cost and widespread implementations of UQ for universal pretrained uMLFFs is a necessary future development.

Table S1: Detailed list of test-set materials (part one) with JARVIS ID, space-group number and symbol, and optB88-vdW band gap rounded to two decimals.

**JID**	**Formula**	**SPG Number**	**SPG Symbol**	**optb88vdw Band Gap (eV)**
JVASP-1002	Si	227	*Fd-3m*	0.73
JVASP-10036	TiO_2	136	$P4_2/mnm$	1.77
JVASP-10037	SnO_2	136	$P4_2/mnm$	0.89
JVASP-1008	Sn	227	*Fd-3m*	0.00
JVASP-1023	Te	152	$P3_121$	0.17
JVASP-1029	Ti	191	*P6/mmm*	0.00
JVASP-103127	$AlInSb_2$	115	$P-4m2$	0.24
JVASP-104	TiO_2	141	$I4_1/amd$	2.02
JVASP-104764	Al_3GaN_4	6	*Pm*	3.51
JVASP-105410	SiGe	216	$F-43m$	0.69
JVASP-10591	ZnS	186	$P6_3mc$	2.10
JVASP-106363	$InGaCu_2Se_4$	82	$I-4$	0.02
JVASP-106686	$ZnHgTe_2$	115	$P-4m2$	0.00
JVASP-1067	Bi_2Se_3	166	$R-3m$	0.32
JVASP-107	SiC	186	$P6_3mc$	2.50
JVASP-10703	Cd_3As_2	224	$Pn-3m$	0.00
JVASP-108770	$InGaSb_2$	115	$P-4m2$	0.00
JVASP-110	$BaTiO_3$	99	*P4mm*	1.75
JVASP-110231	$InGaN_2$	156	*P3m1*	0.22
JVASP-1103	TePb	225	$Fm-3m$	1.12
JVASP-1109	SnS	62	*Pnma*	1.02
JVASP-111005	$SnTe_2Pb$	166	$R-3m$	0.49
JVASP-1112	PbS	225	$Fm-3m$	0.56
JVASP-1115	PbSe	225	$Fm-3m$	0.51
JVASP-113	ZrO_2	14	$P2_1/c$	3.62
JVASP-1174	GaAs	216	$F-43m$	0.09
JVASP-1177	GaSb	216	$F-43m$	0.00
JVASP-1180	InN	186	$P6_3mc$	0.00
JVASP-1183	InP	216	$F-43m$	0.33
JVASP-1186	InAs	216	$F-43m$	0.00
JVASP-1189	InSb	216	$F-43m$	0.00
JVASP-1192	CdSe	216	$F-43m$	0.46
JVASP-1195	ZnO	186	$P6_3mc$	0.97
JVASP-1198	ZnTe	216	$F-43m$	1.06
JVASP-1201	CuCl	216	$F-43m$	0.73
JVASP-1216	Cu_2O	224	$Pn-3m$	0.64
JVASP-1222	UO_2	225	$Fm-3m$	0.00
JVASP-1240	$LiNbO_3$	161	*R3c*	3.34
JVASP-131	SnS_2	164	$P-3m1$	1.22
JVASP-1312	BP	216	$F-43m$	1.52
JVASP-1327	AlP	216	$F-43m$	1.79
JVASP-133719	BAs	216	$F-43m$	1.30
JVASP-1372	AlAs	216	$F-43m$	1.68
JVASP-1408	AlSb	216	$F-43m$	1.32
JVASP-14616	Li	229	$Im-3m$	0.00
JVASP-14968	$TiSi_2$	70	*Fddd*	0.00
JVASP-14970	Si_2Mo	139	*I4/mmm*	0.00
JVASP-149871	$GaAgS_2$	122	$I-42d$	0.89
JVASP-149906	$ZnCdTe_2$	122	$I-42d$	0.68
JVASP-149916	SnTe	225	$Fm-3m$	0.48
JVASP-18983	TiO_2	61	*Pbca*	2.25

Table S2: Detailed list of test-set materials (part two) with JARVIS ID, space-group number and symbol, and optB88-vdW band gap rounded to two decimals.

**JID**	**Formula**	**SPG Number**	**SPG Symbol**	**optb88vdw Band Gap (eV)**
JVASP-1915	InSe	160	$R3m$	0.17
JVASP-19780	Si_2W	139	$I4/mmm$	0.00
JVASP-20092	CdO	225	$Fm-3m$	0.00
JVASP-21211	Se	152	$P3_{121}$	0.90
JVASP-22694	NiO	225	$Fm-3m$	0.00
JVASP-23	CdTe	216	$F-43m$	0.50
JVASP-2376	$ZnSiP_2$	122	$I-42d$	1.43
JVASP-25	Bi_2Te_3	166	$R-3m$	0.35
JVASP-29539	PbI_2	186	$P6_3mc$	2.26
JVASP-30	GaN	186	$P6_3mc$	1.94
JVASP-32	Al_2O_3	167	$R-3c$	6.43
JVASP-34249	HfO_2	225	$Fm-3m$	4.04
JVASP-34674	SiO_2	20	$C222_1$	5.67
JVASP-3510	BiI_3	148	$R-3$	2.37
JVASP-36018	GeC	216	$F-43m$	1.93
JVASP-36408	SnC	216	$F-43m$	0.75
JVASP-36873	BSb	216	$F-43m$	0.95
JVASP-39	AlN	186	$P6_3mc$	4.47
JVASP-41	SiO_2	154	$P3_{221}$	5.99
JVASP-4282	$CrBr_3$	148	$R-3$	1.30
JVASP-43367	HfO_2	61	$Pbca$	4.03
JVASP-5224	HgI_2	137	$P4_2/nmc$	0.97
JVASP-54	MoS_2	194	$P6_3/mmc$	0.92
JVASP-58349	SiO_2	152	$P3_{121}$	5.99
JVASP-62940	BN	194	$P6_3/mmc$	4.46
JVASP-7836	BN	216	$F-43m$	4.81
JVASP-79522	CuO	131	$P4_2/mmc$	0.00
JVASP-8003	CdS	216	$F-43m$	0.99
JVASP-802	Hf	194	$P6_3/mmc$	0.00
JVASP-8082	$SrTiO_3$	221	$Pm-3m$	1.81
JVASP-8118	SiC	186	$P6_3mc$	2.62
JVASP-813	Ag	225	$Fm-3m$	0.00
JVASP-8158	SiC	216	$F-43m$	1.62
JVASP-816	Al	225	$Fm-3m$	0.00
JVASP-8184	GaP	186	$P6_3mc$	1.26
JVASP-825	Au	225	$Fm-3m$	0.00
JVASP-85416	Ag_2S	14	$P2_1/c$	1.11
JVASP-85478	Cu_2S	96	$P4_{32}/12$	0.59
JVASP-8554	$InCuSe_2$	122	$I-42d$	0.01
JVASP-8559	TlBr	221	$Pm-3m$	2.02
JVASP-861	Cr	229	$Im-3m$	0.00
JVASP-867	Cu	225	$Fm-3m$	0.00
JVASP-890	Ge	227	$Fd-3m$	0.00
JVASP-90668	$ZnCu_2SnS_4$	82	$I-4$	0.12
JVASP-91	C	227	$Fd-3m$	4.46
JVASP-9117	FeS_2	205	$Pa-3$	0.43
JVASP-9147	HfO_2	14	$P2_1/c$	4.12
JVASP-9166	B_6As	166	$R-3m$	2.74
JVASP-943	Ni	225	$Fm-3m$	0.00
JVASP-96	ZnSe	216	$F-43m$	1.22
JVASP-963	Pd	225	$Fm-3m$	0.00
JVASP-972	Pt	225	$Fm-3m$	0.00
JVASP-99732	$CdHg_3Te_4$	215	$P-43m$	0.00

Table S3: The percentage of unconverged structural relaxations for bulk, surface, and vacancy calculations for each uMLFF (relaxed using the `ExpCellFilter`). The green highlights which models have the highest convergence rate.

**uMLFF Type**	**Bulk**	**Surface**	**Vacancy**
alignn_ff	7	38	38
chgnet	0	1	0
eqV2_153M_omat	0	15	2
eqV2_31M_omat	0	17	0
eqV2_31M_omat_mp_salex	0	6	0
eqV2_86M_omat	2	18	2
eqV2_86M_omat_mp_salex	0	6	0
mace	0	7	4
mace-alexandria	3	16	2
mace-mpa	0	7	0
matgl	0	5	10
matgl-direct	4	7	13
mattersim	0	5	2
orb-d3-v2	0	1	0
orb-v2	1	0	0
sevennet	0	2	0

Table S4: The mean absolute error (MAE) for lattice constants a, b, c, and volume calculated with each uMLFF type (relaxed using the `ExpCellFilter`), compared to JARVIS-DFT data. The green highlights the best performing models.

**uMLFF Type**	**err_a (Å)**	**err_b (Å)**	**err_c (Å)**	**err_vol (Å^3)**
alignn_ff	0.087	0.102	0.137	9.95
chgnet	0.046	0.049	0.109	3.58
eqV2_153M_omat	0.036	0.040	0.111	2.90
eqV2_31M_omat	0.029	0.032	0.099	3.05
eqV2_31M_omat_mp_salex	0.028	0.030	0.096	3.17
eqV2_86M_omat	0.033	0.040	0.094	3.41
eqV2_86M_omat_mp_salex	0.027	0.030	0.100	3.14
mace	0.035	0.038	0.084	3.00
mace-alexandria	0.081	0.086	0.206	6.05
mace-mpa	0.044	0.046	0.098	3.57
matgl	0.052	0.057	0.128	3.50
matgl-direct	0.044	0.046	0.106	2.88
mattersim	0.031	0.033	0.110	3.18
orb-d3-v2	0.031	0.030	0.048	1.90
orb-v2	0.023	0.025	0.159	3.38
sevennet	0.035	0.038	0.100	3.43

Tutorial

Installation and Setup

To install CHIPS-FF, first clone the repository and set up a conda environment.

Cloning and Setting Up the Environment

```
git clone https://github.com/usnistgov/chipsff
conda env create -f environment.yml -n chipsff
conda activate chipsff
cd chipsff
pip install -e .
```

Input Configuration

CHIPS-FF utilizes a JSON input file that specifies various parameters such as the material identifier, calculator type, and simulation settings. Below is an example input file (`input.json`) for a single material:

Example input.json


```
{
  "jid": "JVASP-1002",
  "calculator_type": "chgnet",
  "chemical_potentials_file": "chemical_potentials.json",
  "properties_to_calculate": [
    "relax_structure",
    "calculate_ev_curve",
    "calculate_formation_energy",
    "calculate_elastic_tensor",
    "run_phonon_analysis",
    "analyze_surfaces",
    "analyze_defects",
    "run_phonon3_analysis",
    "general_melter",
    "calculate_rdf"
  ],
  "bulk_relaxation_settings": {
    "filter_type": "FrechetCellFilter",
    "relaxation_settings": {
      "fmax": 0.05,
      "steps": 200,
      "constant_volume": false
    }
  },
  "phonon_settings": {
    "dim": [2, 2, 2],
    "distance": 0.01
  },
  "use_conventional_cell": false
}
```

Here is an example input file for material interfaces (e.g., `interface_input.json`):

Example interface_input.json

```
{
  "film_id": ["JVASP-1002"],
  "substrate_id": ["JVASP-816"],
  "calculator_type": "alignn_ff",
  "chemical_potentials_file": "chemical_potentials.json",
  "film_index": "1_1_0",
  "substrate_index": "1_1_0",
  "properties_to_calculate": [
    "analyze_interfaces"
  ]
}
```

Running the Simulations

The main `run_chipsff.py` script allows for a command-line interface for executing the simulations. For a single material analysis:

Running Single Material Analysis

```
python run_chipsff.py --input_file input.json
```

For interface analysis:

Running Interface Analysis

```
python run_chipsff.py --input_file interface_input.json
```

Overview of Key Methods

The CHIPS-FF framework includes several key functions:

- `relax_structure()`: Optimizes atomic structures.
- `calculate_formation_energy()`: Computes formation energies using relaxed structures and chemical potential data.
- `calculate_elastic_tensor()`: Evaluates elastic properties.
- `calculate_ev_curve()`: Fits energy-volume curves to determine equilibrium parameters.
- `run_phonon_analysis()`: Performs phonon band structure and thermal property calculations.
- `analyze_defects()` and `analyze_surfaces()`: Analyze defect and surface energies.
- `analyze_defects_from_db()` Analyze defect energies with initial structures directly from the JARVIS-DFT vacancy database.
- `run_phonon3_analysis()`: Calculates third order force constants for thermal conductivity.
- `general_melter()`: Executes MD simulations for melting and quenching processes.
- `analyze_interfaces()`: Conducts interface analysis between film and substrate materials.

References

(1) Behler, J.; Parrinello, M. Generalized neural-network representation of high-dimensional potential-energy surfaces. *Physical review letters* **2007**, *98*, 146401.

(2) Bartók, A. P.; Payne, M. C.; Kondor, R.; Csányi, G. Gaussian approximation potentials: The accuracy of quantum mechanics, without the electrons. *Physical review letters* **2010**, *104*, 136403.

(3) Wood, M. A.; Thompson, A. P. Extending the accuracy of the SNAP interatomic potential form. *The Journal of chemical physics* **2018**, *148*, 241721.

(4) Musaelian, A.; Batzner, S.; Johansson, A.; Sun, L.; Owen, C. J.; Kornbluth, M.; Kozinsky, B. Learning local equivariant representations for large-scale atomistic dynamics. *Nature Communications* **2023**, *14*, 579.

(5) Chen, C.; Ong, S. P. A universal graph deep learning interatomic potential for the periodic table. *Nature Computational Science* **2022**, *2*, 718–728.

(6) Materials Virtual Lab MatGL: Graph Learning for Materials Science. `https://github.com/materialsvirtuallab/matgl`, 2024; Accessed: 2024-10-02.

(7) Wang, M.; Zheng, D.; Ye, Z.; Gan, Q.; Li, M.; Song, X.; Zhou, J.; Ma, C.; Yu, L.; Gai, Y.; Xiao, T.; He, T.; Karypis, G.; Li, J.; Zhang, Z. Deep Graph Library: A Graph-Centric, Highly-Performant Package for Graph Neural Networks. Submitted September 2019. arXiv:1909.01315. https://arxiv.org/abs/1909.01315 (accessed 2024-06-03).

(8) Paszke, A. et al. PyTorch: An Imperative Style, High-Performance Deep Learning Library. Submitted December 2019. arXiv:1912.01703. https://arxiv.org/abs/1912.01703 (accessed 2024-05-02).

(9) Chen, C.; Ye, W.; Zuo, Y.; Zheng, C.; Ong, S. P. Graph Networks as a Universal Machine Learning Framework for Molecules and Crystals. *Chemistry of Materials* **2019**, *31*, 3564–3572.

(10) Choudhary, K.; DeCost, B.; Major, L.; Butler, K.; Thiyagalingam, J.; Tavazza, F.

Unified graph neural network force-field for the periodic table: solid state applications. *Digital Discovery* **2023**, *2*, 346–355.

(11) Choudhary, K.; DeCost, B. Atomistic Line Graph Neural Network for improved materials property predictions. *npj Computational Materials* **2021**, *7*, 185.

(12) Deng, B.; Zhong, P.; Jun, K.; Riebesell, J.; Han, K.; Bartel, C. J.; Ceder, G. CHGNet as a pretrained universal neural network potential for charge-informed atomistic modelling. *Nature Machine Intelligence* **2023**, *5*, 1031–1041.

(13) Batatia, I.; Kovacs, D. P.; Simm, G. N. C.; Ortner, C.; Csanyi, G. MACE: Higher Order Equivariant Message Passing Neural Networks for Fast and Accurate Force Fields. Advances in Neural Information Processing Systems. 2022.

(14) Batatia, I.; Batzner, S.; Kovács, D. P.; Musaelian, A.; Simm, G. N. C.; Drautz, R.; Ortner, C.; Kozinsky, B.; Csányi, G. The Design Space of E(3)-Equivariant Atom-Centered Interatomic Potentials. 2022.

(15) Batatia, I. et al. A foundation model for atomistic materials chemistry. Submitted January 2024. arXiv:2401.00096. https://arxiv.org/abs/2401.00096 (accessed 2024-03-20).

(16) Drautz, R. Atomic cluster expansion for accurate and transferable interatomic potentials. *Phys. Rev. B* **2019**, *99*, 014104.

(17) Dusson, G.; Bachmayr, M.; Csányi, G.; Drautz, R.; Etter, S.; van der Oord, C.; Ortner, C. Atomic cluster expansion: Completeness, efficiency and stability. *Journal of Computational Physics* **2022**, *454*, 110946.

(18) Team, A. MACE-MP: ACE Multi-Physics Framework. `https://github.com/ACEsuit/mace-mp/releases/tag/mace_mpa_0`, 2024; `https://github.com/ACEsuit/mace-mp/releases/tag/mace_mpa_0`, Version 0 release.

(19) Park, Y.; Kim, J.; Hwang, S.; Han, S. Scalable Parallel Algorithm for Graph Neural Network Interatomic Potentials in Molecular Dynamics Simulations. *Journal of Chemical Theory and Computation* **2024**, *20*, 4857–4868.

(20) Batzner, S.; Musaelian, A.; Sun, L.; Geiger, M.; Mailoa, J. P.; Kornbluth, M.; Molinari, N.; Smidt, T. E.; Kozinsky, B. E(3)-equivariant graph neural networks for data-efficient and accurate interatomic potentials. *Nature Communications* **2022**, *13*, 2453.

(21) Yang, H. et al. MatterSim: A Deep Learning Atomistic Model Across Elements, Temperatures and Pressures. Submitted May 2024. arXiv:2405.04967. https://arxiv.org/abs/2405.04967 (accessed 2024-12-10).

(22) Merchant, A.; Batzner, S.; Schoenholz, S. S.; Aykol, M.; Cheon, G.; Cubuk, E. D. Scaling deep learning for materials discovery. *Nature* **2023**, *624*, 80–85.

(23) Takamoto, S. et al. Towards universal neural network potential for material discovery applicable to arbitrary combination of 45 elements. *Nature Communications* **2022**, *13*, 2991.

(24) Microsoft MatterSim: Simulator for Visual Language Navigation. `https://github.com/microsoft/mattersim`, 2024; Accessed: 2024-12-06.

(25) Orbital Materials Orb-Models: Machine Learning Models for Orbital Materials. `https://github.com/orbital-materials/orb-models`, 2024; Accessed: 2024-10-02.

(26) Neumann, M.; Gin, J.; Rhodes, B.; Bennett, S.; Li, Z.; Choubisa, H.; Hussey, A.; Godwin, J. Orb: A Fast, Scalable Neural Network Potential. Submitted October 2024. arXiv:2410.22570. https://arxiv.org/abs/2410.22570 (accessed 2024-11-01).

(27) Orbital Materials Introducing the Orb AI-Based Interatomic Potential. `https://www.orbitalmaterials.com/post/`

technical-blog-introducing-the-orb-ai-based-interatomic-potential, 2024; Accessed: 2024-10-02.

(28) Riebesell, J.; Goodall, R.; Benner, P.; Chiang, Y.; Deng, B.; Lee, A.; Jain, A.; Persson, K. Matbench Discovery: A Benchmark for AI-Accelerated Materials Discovery. https://matbench-discovery.materialsproject.org/preprint, 2024; Accessed: 2024-10-02.

(29) Sanchez-Gonzalez, A.; Godwin, J.; Pfaff, T.; Ying, R.; Leskovec, J.; Battaglia, P. W. Learning to Simulate Complex Physics with Graph Networks. Submitted February 2020. arXiv:2002.09405. https://arxiv.org/abs/2002.09405 (accessed 2024-09-27).

(30) Gilmer, J.; Schoenholz, S. S.; Riley, P. F.; Vinyals, O.; Dahl, G. E. Neural Message Passing for Quantum Chemistry. Submitted April 2017. arXiv:1704.01212. https://arxiv.org/abs/1704.01212 (accessed 2025-09-02).

(31) Barroso-Luque, L.; Shuaibi, M.; Fu, X.; Wood, B. M.; Dzamba, M.; Gao, M.; Rizvi, A.; Zitnick, C. L.; Ulissi, Z. W. Open Materials 2024 (OMat24) Inorganic Materials Dataset and Models. Submitted October 2024. arXiv:2410.12771. https://arxiv.org/abs/2410.12771 (accessed 2024-11-01).

(32) Liao, Y.-L.; Wood, B.; Das, A.; Smidt, T. EquiformerV2: Improved Equivariant Transformer for Scaling to Higher-Degree Representations. Submitted June 2023. arXiv:2306.12059. https://arxiv.org/abs/2306.12059 (accessed 2024-11-01).

(33) Hjorth Larsen, A. et al. The atomic simulation environment—a Python library for working with atoms. *Journal of Physics: Condensed Matter* **2017**, *29*, 273002.

(34) Wines, D.; Gurunathan, R.; Garrity, K. F.; DeCost, B.; Biacchi, A. J.; Tavazza, F.; Choudhary, K. Recent progress in the JARVIS infrastructure for next-generation data-driven materials design. *Applied Physics Reviews* **2023**, *10*, 041302.

(35) Choudhary, K. et al. The joint automated repository for various integrated simulations (JARVIS) for data-driven materials design. *npj Computational Materials* **2020**, *6*, 173.

(36) Togo, A.; Chaput, L.; Tanaka, I. Distributions of phonon lifetimes in Brillouin zones. *Phys. Rev. B* **2015**, *91*, 094306.

(37) Togo, A.; Chaput, L.; Tadano, T.; Tanaka, I. Implementation strategies in phonopy and phono3py. *J. Phys. Condens. Matter* **2023**, *35*, 353001.

(38) Jochym, P. T. Module for calculating elastic tensor of crystals. `https://github.com/jochym/Elastic/`, 2022.

(39) Jochym, P. T.; Parlinski, K.; Sternik, M. TiC lattice dynamics from ab initio calculations. *The European Physical Journal B - Condensed Matter and Complex Systems* **1999**, *10*, 9–13.

(40) Jochym, P. T.; Parlinski, K. Ab initio lattice dynamics and elastic constants of ZrC. *The European Physical Journal B - Condensed Matter and Complex Systems* **2000**, *15*, 265–268.

(41) Choudhary, K.; Garrity, K. F. InterMat: accelerating band offset prediction in semiconductor interfaces with DFT and deep learning. *Digital Discovery* **2024**, *3*, 1365–1377.

(42) Zur, A.; McGill, T. C. Lattice match: An application to heteroepitaxy. *Journal of Applied Physics* **1984**, *55*, 378–386.

(43) Qi, J.; Ko, T. W.; Wood, B. C.; Pham, T. A.; Ong, S. P. Robust training of machine learning interatomic potentials with dimensionality reduction and stratified sampling. *npj Computational Materials* **2024**, *10*, 43.

(44) Grimme, S.; Antony, J.; Ehrlich, S.; Krieg, H. A consistent and accurate ab initio parametrization of density functional dispersion correction (DFT-D) for the 94 elements H-Pu. *The Journal of Chemical Physics* **2010**, *132*, 154104.

(45) Schmidt, J.; Cerqueira, T. F.; Romero, A. H.; Loew, A.; Jäger, F.; Wang, H.-C.; Botti, S.; Marques, M. A. Improving machine-learning models in materials science through large datasets. *Materials Today Physics* **2024**, *48*, 101560.

(46) Perdew, J. P.; Ruzsinszky, A.; Csonka, G. I.; Vydrov, O. A.; Scuseria, G. E.; Constantin, L. A.; Zhou, X.; Burke, K. Restoring the Density-Gradient Expansion for Exchange in Solids and Surfaces. *Phys. Rev. Lett.* **2008**, *100*, 136406.

(47) Team, H. 2D Universal Force Field CPU Model (Alexandria v2). `https://github.com/hyllios/utils/blob/main/models/alexandria_v2/mace/2D_universal_force_field_cpu.model`, 2024; `https://github.com/hyllios/utils/blob/main/models/alexandria_v2/mace/2D_universal_force_field_cpu.model`, Model file available at GitHub.

(48) Team, F.-C. OMAT24 Model. `https://huggingface.co/fairchem/OMAT24`, 2024; `https://huggingface.co/fairchem/OMAT24`, Accessed: 2024-10-22.

(49) Bitzek, E.; Koskinen, P.; Gähler, F.; Moseler, M.; Gumbsch, P. Structural Relaxation Made Simple. *Phys. Rev. Lett.* **2006**, *97*, 170201.

(50) Atomic Simulation Environment (ASE) Developers ASE Filters Documentation. `https://wiki.fysik.dtu.dk/ase/ase/filters.html`, 2025; Accessed: March 12, 2025.

(51) Chaput, L.; Togo, A.; Tanaka, I.; Hug, G. Phonon-phonon interactions in transition metals. *Phys. Rev. B* **2011**, *84*, 094302.

(52) Togo, A.; Chaput, L.; Tanaka, I.; Hug, G. First-principles phonon calculations of thermal expansion in Ti_3SiC_2, Ti_3AlC_2, and Ti_3GeC_2. *Phys. Rev. B* **2010**, *81*, 174301.

(53) Choudhary, K.; Sumpter, B. G. Can a deep-learning model make fast predictions of vacancy formation in diverse materials? *AIP Advances* **2023**, *13*, 095109.

(54) Kresse, G.; Furthmüller, J. Efficient Iterative Schemes for ab initio Total-energy Calculations Using a Plane-wave Basis Set. *Phys. Rev. B* **1996**, *54*, 11169–11186.

(55) Kresse, G.; Joubert, D. From Ultrasoft Pseudopotentials to the Projector Augmented-wave Method. *Phys. Rev. B* **1999**, *59*, 1758–1775.

(56) Nose, S. A unified formulation of the constant temperature molecular dynamics methods. *The Journal of Chemical Physics* **1984**, *81*, 511–519.

(57) Shuichi, N. Constant Temperature Molecular Dynamics Methods. *Progress of Theoretical Physics Supplement* **1991**, *103*, 1–46.

(58) Hoover, W. G. Canonical dynamics: Equilibrium phase-space distributions. *Phys. Rev. A* **1985**, *31*, 1695–1697.

(59) Van de Walle, C. G.; Martin, R. M. Theoretical study of band offsets at semiconductor interfaces. *Phys. Rev. B* **1987**, *35*, 8154–8165.

(60) Di Liberto, G.; Pacchioni, G. Band offset in semiconductor heterojunctions. *Journal of Physics: Condensed Matter* **2021**, *33*, 415002.

(61) Lakshminarayanan, B.; Pritzel, A.; Blundell, C. Simple and Scalable Predictive Uncertainty Estimation using Deep Ensembles. Submitted December 2016. arXiv:1612.01474. https://arxiv.org/abs/1612.01474 (accessed 2025-03-05).

(62) Amini, A.; Schwarting, W.; Soleimany, A.; Rus, D. Deep evidential regression. Advances in Neural Information Processing Systems. *2020-Decem (NeurIPS)* **2020**, 1–19.

(63) Nix, D. A.; Weigend, A. S. Estimating the mean and variance of the target probability distribution. Proceedings of 1994 ieee international conference on neural networks (ICNN'94). 1994; pp 55–60.

(64) Zhu, A.; Batzner, S.; Musaelian, A.; Kozinsky, B. Fast uncertainty estimates in deep learning interatomic potentials. *The Journal of Chemical Physics* **2023**, *158*, 164111.

(65) Tan, A. R.; Urata, S.; Goldman, S.; Dietschreit, J. C. B.; Gómez-Bombarelli, R. Single-model uncertainty quantification in neural network potentials does not consistently outperform model ensembles. *npj Computational Materials* **2023**, *9*, 225.

(66) Vita, J. A.; Samanta, A.; Zhou, F.; Lordi, V. LTAU-FF: Loss Trajectory Analysis for Uncertainty in Atomistic Force Fields. Submitted February 2024. arXiv:2402.00853. https://arxiv.org/abs/2402.00853 (accessed 2025-03-05).

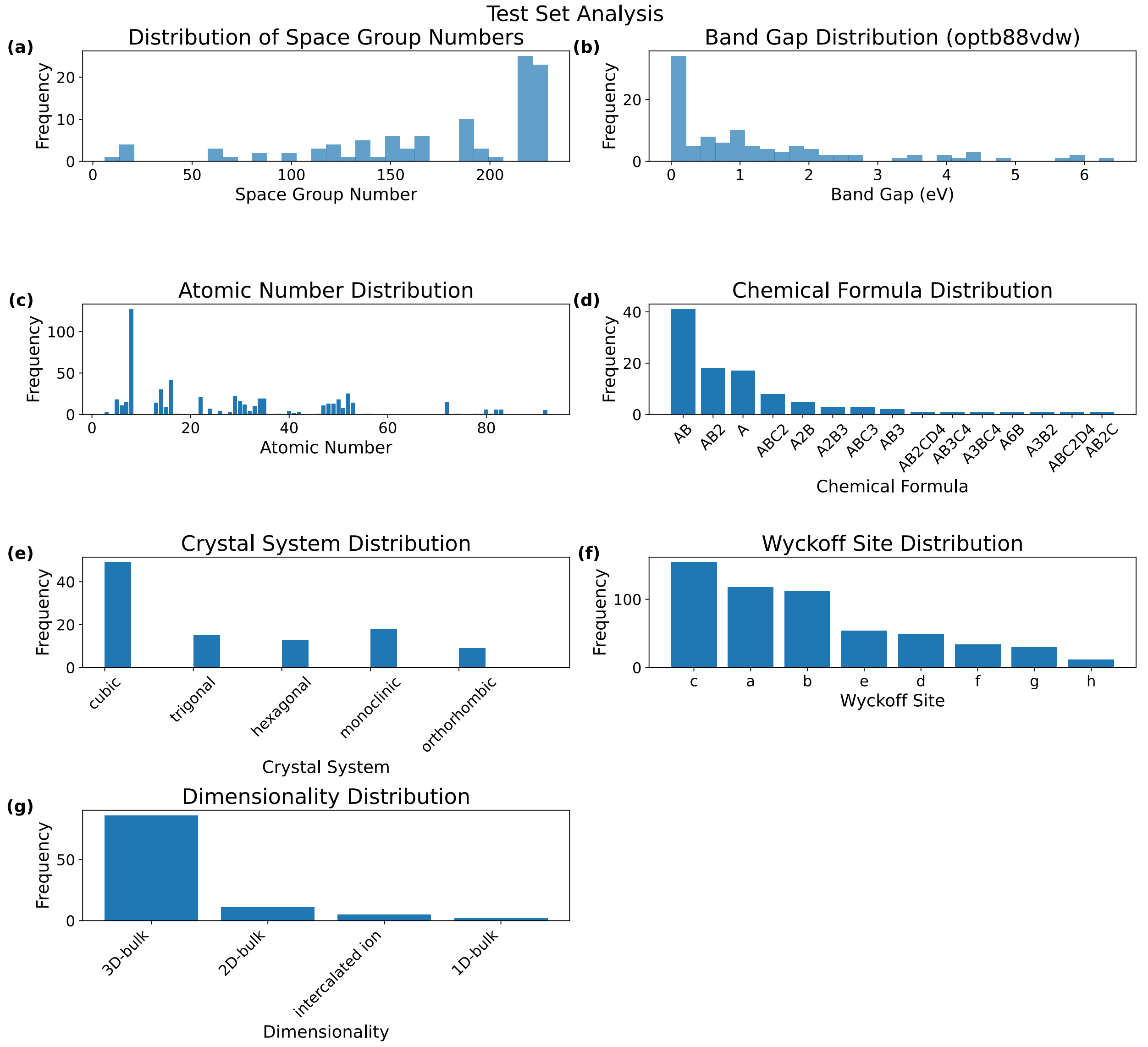

Fig. S1: The data distribution of the 104 materials in the test set for a) space group number, b) band gap (vdW-DF-optB88), c) atomic number, d) chemical formula, e) crystal system, f) Wyckoff site, g) dimensionality.
